# High postoperative blood levels of macrophage migration inhibitory factor are associated with less organ dysfunction in patients after cardiac surgery

**DOI:** 10.1186/cc11078

**Published:** 2012-03-20

**Authors:** C Stoppe, G Grieb, D Simons, R Rossaint, J Bernhagen, S Rex

**Affiliations:** 1 University Hospital of the RWTH, Aachen, Germany; 2 University Hospital Gasthuisberg, KU Leuven, Belgium

## Introduction

Macrophage migration inhibitory factor (MIF) is a structurally unique inflammatory cytokine [1] that exerts protective effects during ischemia and reperfusion [2]. We hypothesized that elevated MIF levels in the early postoperative time course might be inversely associated with postoperative organ dysfunction as assessed by SAPS II and SOFA score in patients after cardiac surgery.

## Methods

Fifty-two cardiac surgical patients (mean age (± SD) 67 ± 10 years; EuroSCORE: 7 (2 to 11)) were enrolled in this monocenter, prospective, observational study. Serum levels of MIF and clinical data were obtained after induction of anesthesia, at admission to the ICU, 4 hours thereafter and at the first and second postoperative day (POD). Patient outcome was assessed using the SAPS II at POD1 and SOFA score for the first 3 days of the eventual ICU stay.

## Results

MIF_AUC, the computed area under the curve of MIF serum levels from admission until POD1, was inversely correlated with SAPS II and SOFA score on POD1 (Table 1). MIF at admission (*r *= 0.296; *P *= 0.041) and MIF at 4 hours (*r *= 0.367; *P *= 0.012) correlated inversely with the paO_2_/FiO_2 _ratios at POD1. Moreover, postoperative MIF values were inversely correlated with SAPS II (*r *= 0.528; *P *= 0.044) and SOFA scores during the early postoperative stay (Table 1). In addition, MIF values on POD1 were related to the calculated Cardiac Power Index (*r *= 0.420; *P *= 0.009).

**Table 1:** 

MIF level	SOFA 1. POD	SOFA 2. POD	SOFA 3. POD
ICU admission	*r *= -0.2; *P *= 0.18	*r *= -0.4; *P *= 0.11	*r *= -0.6; *P *= 0.05
4 hours later	*r *= -0.4; *P *= 0.40	*r *= -0.5; *P *= 0.05	*r *= -0.8; *P *= 0.01
MIF_AUC	*r *= -0.4; *P *= 0.01	*r *= -0.2; *P *= 0.55	*r *= -0.4; *P *= 0.19
1. POD	*r *= -0.3; *P *= 0.08	*r *= -0.6; *P *= 0.03	*r *= -0.7; *P *= 0.02

## Conclusion

Elevated postoperative MIF levels are inversely correlated with organ dysfunction in patients after cardiac surgery.
